# Neuroendocrine effects of the duper mutation in Syrian hamsters: a role for *Cryptochrome 1*

**DOI:** 10.3389/fphys.2024.1351682

**Published:** 2024-02-20

**Authors:** Emily N. C. Manoogian, Michael Seifu Bahiru, Emily Jue Wang, Mary Holder, Eric L. Bittman

**Affiliations:** Department of Biology and Program in Neuroscience, University of Massachusetts, Amherst, MA, United States

**Keywords:** circadian rhythms, LH surge, *Cryptochrome* 1, melatonin, photoperiod, duper

## Abstract

Molecular and physiological determinants of the timing of reproductive events, including the pre-ovulatory LH surge and seasonal fluctuations in fertility, are incompletely understood. We used the *Cryptochrome 1*-deficient duper mutant to examine the role of this core circadian clock gene in Syrian hamsters. We find that the phase of the LH surge and its stability upon shifts of the light: dark cycle are altered in duper mutants. The intensity of immunoreactive PER1 in GnRH cells of the preoptic area peaks earlier in the day in duper than wild type hamsters. We note that GnRH fibers coursing through the suprachiasmatic nucleus (SCN) contact vasopressin- and VIP-immunoreactive cells, suggesting a possible locus of circadian control of the LH surge. Unlike wild types, duper hamsters do not regress their gonads within 8 weeks of constant darkness, despite evidence of melatonin secretion during the subjective night. In light of the finding that the duper allele is a stop codon in *Cryptochrome 1*, our results suggest important neuroendocrine functions of this core circadian clock gene.

## Introduction

Neuroendocrine function is governed by circadian clocks. As the transcriptional-translational feedback loops (TTFLs) that generate cell-autonomous circadian oscillations are elucidated, the role of specific genes and their protein products in reproductive mechanisms may be clarified. These oscillators run in multiple neural and endocrine structures, and circadian rhythms may control ovulation and seasonal breeding at multiple loci (Miller et al., 2006; Chu et al., 2013; Bittman, 2019).

Mutations of clock components impact the estrous cycle and photoperiodic responses in Syrian hamsters. The *tau* mutation, a gain of function of casein kinase 1e that leads to hyperphosphorylation of PER proteins, abbreviates the negative arm of the TTFL and thus shortens circadian period (Lowrey et al., 2000; Gallego and Virshup, 2007; Meng et al., 2008; Maywood et al., 2014). The free running period of LH surges induced by estradiol treatment of ovariectomized hamsters is reduced to 20 h in *tau* homozygotes (Lucas et al., 1999). Seasonal reproduction is also affected by the *tau* mutation, as both the generation of and the response to pineal melatonin signals is altered (Stirland et al., 1995, 1996a&b). Yet the mechanisms that control the timing and stability of the LH surge and mediate photoperiodic time measurement are incompletely understood.

Duper is a recessive mutation in hamsters, in which deletion of a single base in exon 4 of *Cry1* leads to a frame shift and a stop codon (Lee et al., 2022). The deficiency in CRY1 shortens the free-running period of locomotor rhythms, expands the range of entrainment, amplifies phase shifting responses to acute light pulses in constant darkness, and speeds re-entrainment of locomotor activity rhythms upon shifts of the light:dark cycle (Krug et al., 2011; Monecke et al., 2011; Bittman, 2014; Sisson et al., 2022). The neuroendocrine impact of duper is unknown. In order to address this gap, we examined the entrained phase of the LH surge. We then subjected hamsters to shifts of the light: dark (LD) cycle in order to determine the reproductive consequences of duper in a jet lag paradigm. Finally, we asked whether duper alters reproductive responses to light deprivation by subjecting hamsters to long-term exposure to constant darkness (DD).

## Materials and methods

### General

Wild type hamsters (*Mesocricetus auratus*) of the Lakeview LVG strain were obtained commercially (Charles River, Wilmington MA) or bred in our laboratory from that stock. Animals were allowed *ad libitum* access to food and water throughout these studies. Duper hamsters were derived from the original super duper animals by crossing through 2 generations to Lakeview wild types, as previously described (Monecke et al., 2011). As the genetic identity of the duper allele was not yet known at the time of these experiments, duper mutants used in these experiments were identified by phenotype: free-running period was approximately 23 h, and they descended from duper mutants in which restriction digest mapping of their genomic DNA (Lowrey et al., 2000) confirmed absence of the *tau* mutation. All experiments were approved by the Institutional Animal Care and Use Committee (IACUC) of the University of Massachusetts at Amherst.

To record the rhythm of locomotor activity, adult hamsters were individually housed in activity wheel-equipped cages. Behavioral rhythms were analyzed using ClockLab software (Actimetrics, Wilmette, IL) as previously described (Krug et al., 2011). In order to confirm phenotype, free running circadian period (τ_DD_) was assessed by maintaining animals in constant darkness for approximately 10 days before the procedures described below were performed. Wild-type hamsters displayed τ_DD_ of ∼ 23.9 h and duper hamsters displayed free-running period of ∼ 22.9 h.

#### Experiment 1

The estrous cycles of adult female hamsters (49 duper, 52 wt) were tracked by daily vaginal smears (Kent et al., 1968) while they were maintained in 14L:10D. Groups of hamsters that showed at least 3 consecutive estrous cycles (as marked by a discharge on the morning of every fourth day) were deeply anesthetized with an overdose of pentobarbital (80 mg/kg) at 2 h intervals between ZT3 and ZT 20 (Table 1). Blood was withdrawn by cardiac puncture and stored overnight at 4C. Serum was harvested and assayed for LH. Immediately after blood sampling, hamsters were transcardially perfused with 100 mL 0.1 M phosphate buffer followed by 300 mL 4% paraformaldehyde. Brains were postfixed overnight, transferred to 20% sucrose for 2 days, and sectioned in a 1-in-4 series at 40 µm. Sections were stored in cryoprotectant (Watson et al., 1986) until immunostaining.

**TABLE 1:** GnRH neurons analyzed in Experiment 1.

Time	# WT animals	# Duper animals	# WT cells	# Duper cells	# WT images	#Duper images
ZT2	5	4	165	126	29	23
ZT4	5	4	210	100	28	18
ZT6	4	6	86	146	14	29
ZT8	5	5	180	141	20	23
ZT10	4	5	65	194	12	17
ZT12	5	3	170	129	24	16
ZT14	6	3	228	92	30	16
ZT16	4	2	174	52	32	11
ZT18	3	3	131	120	18	20

#### Experiment 2

**FIGURE 1:**
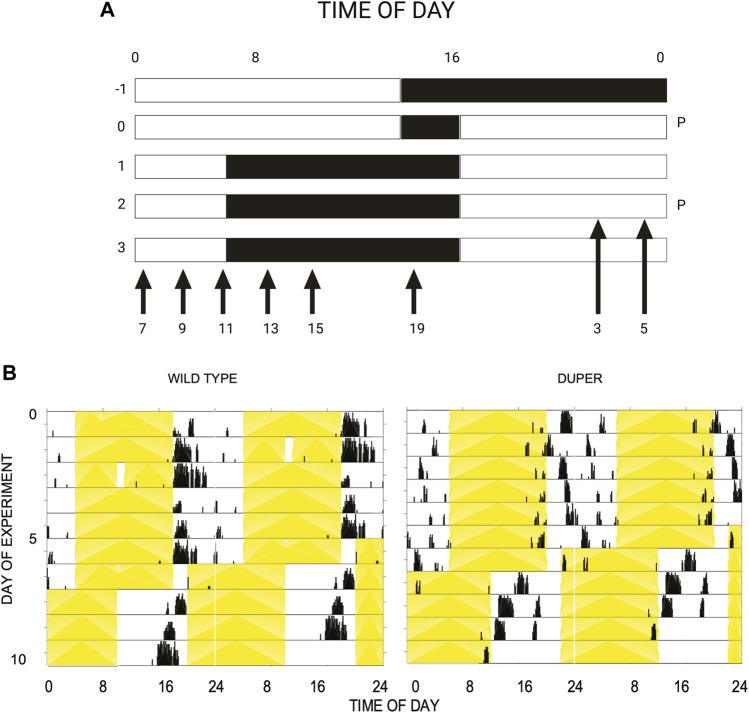
**(A)** Design of experiment 2. Female hamsters housed in 14L:10D cycle were subjected to an acute phase advance on proestrus, by way of shortening dark phase. Starting on the third day after phase advance, animals were sacrificed at time points (ZT) spanning the next anticipated proestrus, as indicated by arrows. The light and dark phases are indicated by the white and black background, respectively. Abbreviation: P: proestrus. **(B)** Double-plotted actograms of representative female wild type (left) and duper (right) hamsters subjected to an acute 8 h phase advance on day 5. Yellow shading indicates light phase. Note that duper hamster entrains with positive phase angle (activity begins 2-3 h before lights out), and re-gains the new phase more rapidly than wt upon shift of the LD cycle.

The latency to re-entrain behavioral rhythms and LH surges was assessed. Female hamsters were maintained in 14L:10D for at least 3 weeks, during which estrous cycles and locomotor activity patterns were tracked as in *Experiment 1*. On the day of proestrus, hamsters were subjected to an 8 hour advance of the LD cycle by shortening the dark phase to 2 hours (Figure 1A). Groups of hamsters (*n* = 37 duper, 50 wt) were deeply anesthetized at 2 hour intervals beginning at ZT3 on the third day after the shift. Sampling spanned the next predicted proestrus, starting from ZT 3 until ZT 15 and at ZT 19 and ZT 23. Blood was collected as described above.

#### Experiment 3

Adult male (*n* = 6 duper, 6 wt) and female (7 duper, 4 wt) hamsters were housed in running wheel cages. They were transferred from 14L:10D to DD for 8 weeks. Constant darkness was interrupted during the subjective day (approximately CT6) at 3 weeks intervals for no longer than 15’ in order to provide freshly bedded cages (Bed-o-cob, Andersons) and to replenish food and water as required for husbandry considerations. These interruptions did not alter locomotor phase, and exposure to light during the circadian dead zone has been established not to photostimulate the gonads (Elliott et al., 1972; Elliott, 1976). At the end of the experiment, hamsters were deeply anesthetized with pentobarbital during the late subjective night. Blood was withdrawn by cardiac puncture and the gonads and uteri were weighed and processed for histological analysis. In order to assess melatonin secretion in wt and duper hamsters, additional male hamsters maintained in 14L:10D were deeply anesthetized at ZT 18–20 (*n* = 5 wt, 5 duper) or ZT 5–8 (*n* = 3 wt) and blood was withdrawn by cardiac puncture. Serum was harvested the following day and assayed for melatonin using ELISA.

### Immunocytochemistry

We analyzed PER1-ir and BMAL1-ir in GnRH cells of 27 duper and 27 wild type hamsters in a single ICC run. 4–6 sections containing preoptic area (POA) and anterior hypothalamus were chosen for each hamster. In the course of this work, we detected GnRH fibers coursing through the SCN region. In order to determine whether day of the estrous cycle influences GnRH staining in the SCN region, additional hamsters were perfused on metestrus at ZT8.5. To examine the possibility that GnRH fiber distribution in this region is sexually differentiated we perfused 4 male hamsters at this time of day. For comparative purposes, we also perfused female mice at ZT12 on the day of proestrus for examination of GnRH immunostaining in the SCN region.

Crypoprotected sections were rinsed in PBS and blocked as previously described (Manoogian et al., 2018). Preoptic sections were stained using rabbit anti-GnRH (1:2,500; LR-1, a generous gift of Robert Benoit), goat anti-Per1 (1:200, Bethyl) and guinea pig anti-Bmal1 (1:20,000, a gift of David R. Weaver; LeSauter et al., 2012). Sections at the level of the SCN were triple labeled for GnRH, AVP and VIP using LR-1, mouse anti-neurophysin (1:50; specific for AVP neurons; a gift of Dr. Harold Gainer) and guinea pig anti-VIP (Peninsula T-5050), respectively. Subsequently, anti-VIP sold under the same catalog number yielded unsatisfactory results. Thus we stained additional sections for GnRH using a guinea pig antibody (1:5,000; a gift of Dr. Erik Hrabovsky) in combination with rabbit anti-VIP (1:10,000, Immunostar) or with LR-1 rabbit anti-GnRH in combination with guinea pig anti-AVP (1:2,000, Peninsula). Second antibodies (Alexa 488 anti guinea pig, Cy3 anti-rabbit, and Cy5 anti-goat or anti-mouse, all raised in donkey) were obtained from Jackson Laboratories (West Grove, PA) and used at a dilution of 1:300. At the completion of incubation in secondary antibodies, tissue sections were rinsed 4X in 0.1 M PB and mounted onto subbed slides. When dry, slides were cover-slipped using Aqua-Poly (Polysciences, Warrington, PA, 18606-20) and stored and protected from light.

Sections were imaged at 10X and 20X using a Zeiss LSM 710 Confocal Microscope with the same settings for laser, gain, and intensity. The standard excitation wavelengths of Cy3 (550 nm), Alexa488 (488 nm), and Cy5 (650 nm) were used to capture GnRH, BMAL1, and PER1 fluorescently-labeled secondary antibodies, respectively. FIJI (Schindelin et al., 2012; http://fiji.sc/Fiji) and scikit-image for Python were used for all image analysis. For quantification of BMAL1 and PER1 colocalization within GnRH cells, maximum intensity projections of 3D stacks were taken for conversion to 2D images. Images were pre-processed by applying a Gaussian filter and performing adaptive histogram equalization. This allowed quantification of the percentage of GnRH cells that contained the protein product of each of the two clock genes above threshold for detection. We applied the watershed algorithm to distance transformed images to segment GnRH cells and quantified the intensity of blue (PER1) and green (BMAL1) channels within the segmented sections. The fluorescence intensity of PER1 and BMAL1 expression in each population of GnRH cells was calculated for each time and genotype condition. This provided a quantitative estimate of expression of each clock gene within those GnRH cells that co-expressed itCode used to compute this is available upon request. The NeuronJ plugin for FIJI (available from ImageScience) was used to manually trace and measure the lengths of immunostained axons within SCN sections.

### Hormone assays

A well-established ultrasensitive ELISA was used to measure serum LH concentrations (Steyn et al., 2013). Briefly, a 96-well high-affinity binding microplate (650101; Greiner Bio-One) was coated with 50 μL of capture antibody (monoclonal antibody, anti-bovine LH beta subunit, 518B7; Dr. Janet Roser, University of California, Davis) at a final dilution of 1:1,000 (in 1X PBS). Wells were incubated with 200 μL of blocking buffer and hormone levels were determined using AFP- 5306A (NIDDK-NHPP) as standard. Polyclonal rabbit LH antiserum, (AFP240580Rb) was used at a final dilution of 1:10,000 to detect bound LH, and secondary horseradish peroxidase-conjugated goat anti-rabbit (D048701-2; DakoCytomation) was detected with ABCAM high sensitivity TMB (Cat #ab171523). The reaction was stopped by the addition of Abcam Cat#ab171529. Absorbance was read at a wavelength of 490 nm using Optima polar Star software. The concentration of LH in whole blood samples was determined by interpolating the OD values of unknowns against a nonlinear regression of the LH standard curve. The average intra-assay coefficient of variation was 9.6%, and the inter-assay coefficient of variation was 5.8%. The profile of the hamster LH surge documented by this ELISA is essentially identical to that obtained through use of standard radioimmunoassays (Norman and Spies, 1974).

Melatonin was extracted from serum collected by cardiac sampling of deeply anesthetized hamsters that had been maintained in DD for 8 weeks or kept in 14L:10D at ZT18-20. Aliquots (200µl) of serum were added to C18 columns, washed with 2 × 1 mL of 10% MeOH followed by 1 mL hexane, eluted with MeOH, and evaporated to dryness. Melatonin concentrations were determined in duplicate using a commercial ELISA (MLTN-96, Novolytix, Witterswil, Switzerland).

### Statistical analysis

Due to the small sample sizes (*n* = 3–8/genotype/time point), we used the non-parametric Kruskal-Wallis test with Dunn’s multiple comparisons test to assess effects of genotype and ZT on serum LH and melatonin, and on ICC data. Further assessment of genotype effects on the LH surge, including area under the curve and fold change was done using the Mann-Whitney test. All data were analyzed and plotted using GraphPad Prism statistical software. Results were considered significant at *p* < 0.05.

Two quantification approaches were used to characterize the rhythms in PER1 and BMAL1 immunoreactivity in GnRH cells. The first assesses the proportion of GnRH neurons with a reported intensity of 2 standard deviations above the mean (background) for either PER1 or BMAL1. This was computed for each section and then aggregated for each animal. The second approach reports the mean intensity of either PER1 or BMAL1 across all the GnRH cells identified in a particular section and aggregated for each animal.

## Results

### Experiment 1

#### Timing of the LH surge in duper mutant and wild type hamsters

Among hamsters maintained in 14L:10D, the LH surge occurred significantly earlier on the day of proestrus in duper mutants than in wild types. The effect of genotype on the time of the LH surge was comparable to the change in the entrained phase of locomotor activity onset (Figure 1B).

**FIGURE 2:**
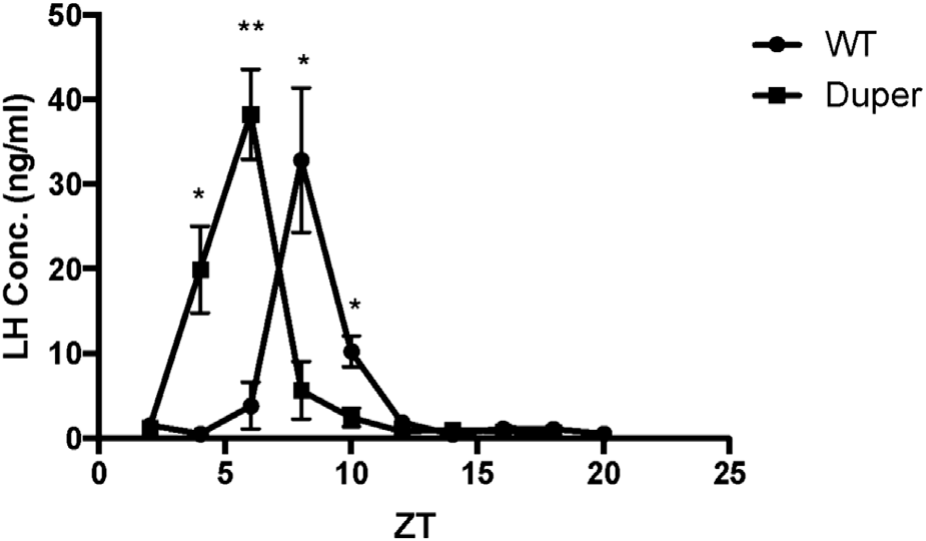
The LH surge occurs earlier in duper than in wt hamsters entrained to 14L:10D. Symbols represent mean (+SEM) concentrations in serum of 5-6 animals collected at each zeitgeber time (ZT). *, statistically significant difference (*p* < 0.001) in LH concentrations between genotypes at the indicated ZT.

Wild-type hamsters demonstrated a robust and temporally limited LH surge, with all 6 animals sampled at ZT8 exhibiting LH concentration greater than 6 ng/mL (Figure 2). LH levels returned to baseline by ZT 12 and remained low for the duration of the night. Mutant duper hamsters demonstrated an altered LH surge profile, with 4 of 5 duper hamsters demonstrating elevated (>6 ng/mL) LH concentration at ZT 4, and all 6 duper hamsters demonstrating LH levels greater than 6 ng/mL at ZT 6 (Figure 2). By the time of the onset of the LH surge in wild-type hamsters at ZT8, LH levels of duper mutants were close to or at baseline (Figure 2; Supplementary Figure 1). Upon finding a significant genotype effect across all sampling times [H (18) = 63.14, *p* < 0.0001; Kruskal-Wallis test], a Dunn’s test was performed to analyze effects at specific phases. This revealed significantly higher LH levels at ZT 4 and ZT 6 in duper hamsters. At ZT 8, wild type hamsters displayed significantly higher LH levels compared to duper hamsters (31.38 ± 5.98 ng/mL, *n* = 6 vs. 5.48 ± 2.78 ng/mL, *n* = 6) (Z = 7.00, *p* < 0.0001). LH fold change, representing the induced LH surge peak to baseline ratio where baseline is defined as the mean of all time points where LH concentration was <5 ng/mL, was significantly higher in duper (40.37 ± 10.52) compared to wild-type hamsters (2.37 ± 0.77) at ZT 6 (U = 0, *p* = 0.04). The total amount of LH released, defined as the area under the curve for each surge, did not differ between wild-type and duper hamsters.

#### Timing of Per1 and Bmal1 expression in GnRH neurons of duper and wt hamsters

**FIGURE 3:**
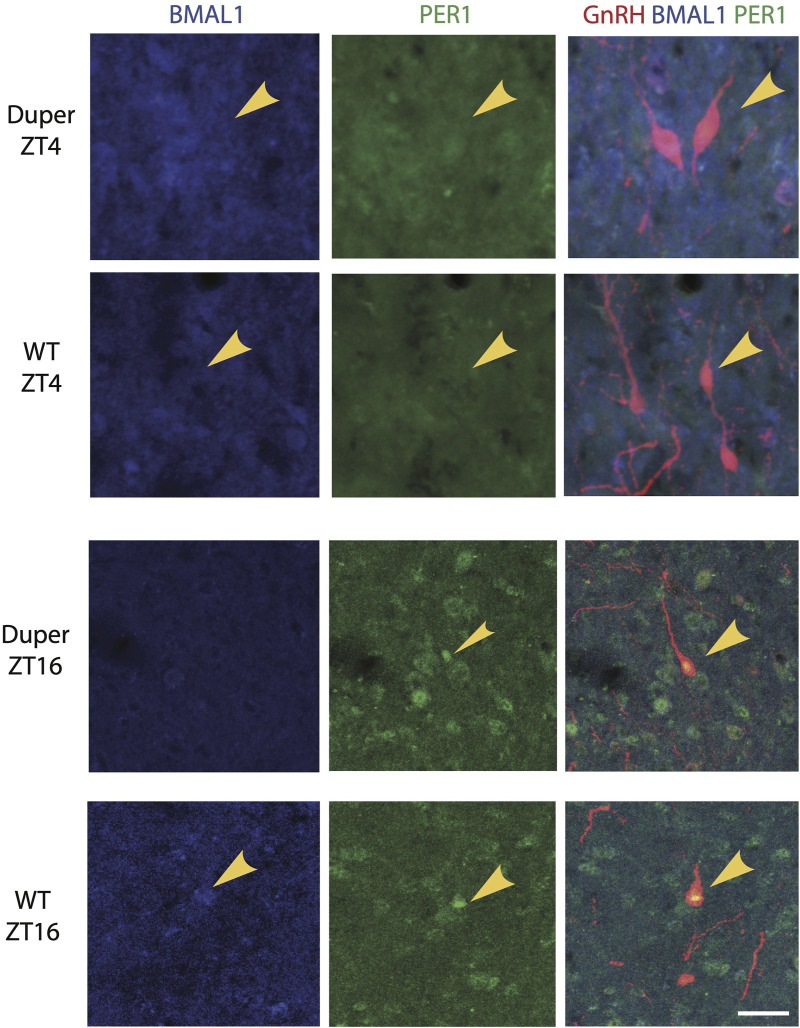
BMAL1 and PER1 proteins are co-localized with GnRH in a time-of- day dependent manner in both wild type and duper Syrian hamsters. Representative triple-label immunocytochemical staining for GnRH, PER1, and BMAL1 are shown in 40 μm sections of preoptic area from wild-type and duper hamsters. Arrows mark BMAL1-positive (blue) GnRH cells at ZT 4 and PER1-positive (green) GnRH cells at ZT 16. Scale bar = 50 μm.

In order to examine the effect of the duper mutation on the timing of activation of GnRH cells, we performed triple-label immunocytochemistry for PER1, BMAL1, and GnRH on proestrus. In both wild-type and duper hamsters, BMAL1 and PER1 proteins were colocalized with GnRH in a time-of-day dependent manner, with higher BMAL-ir1 during the day and PER1-ir peaking during the night (Figure 3; Table 1).

**FIGURE 4:**
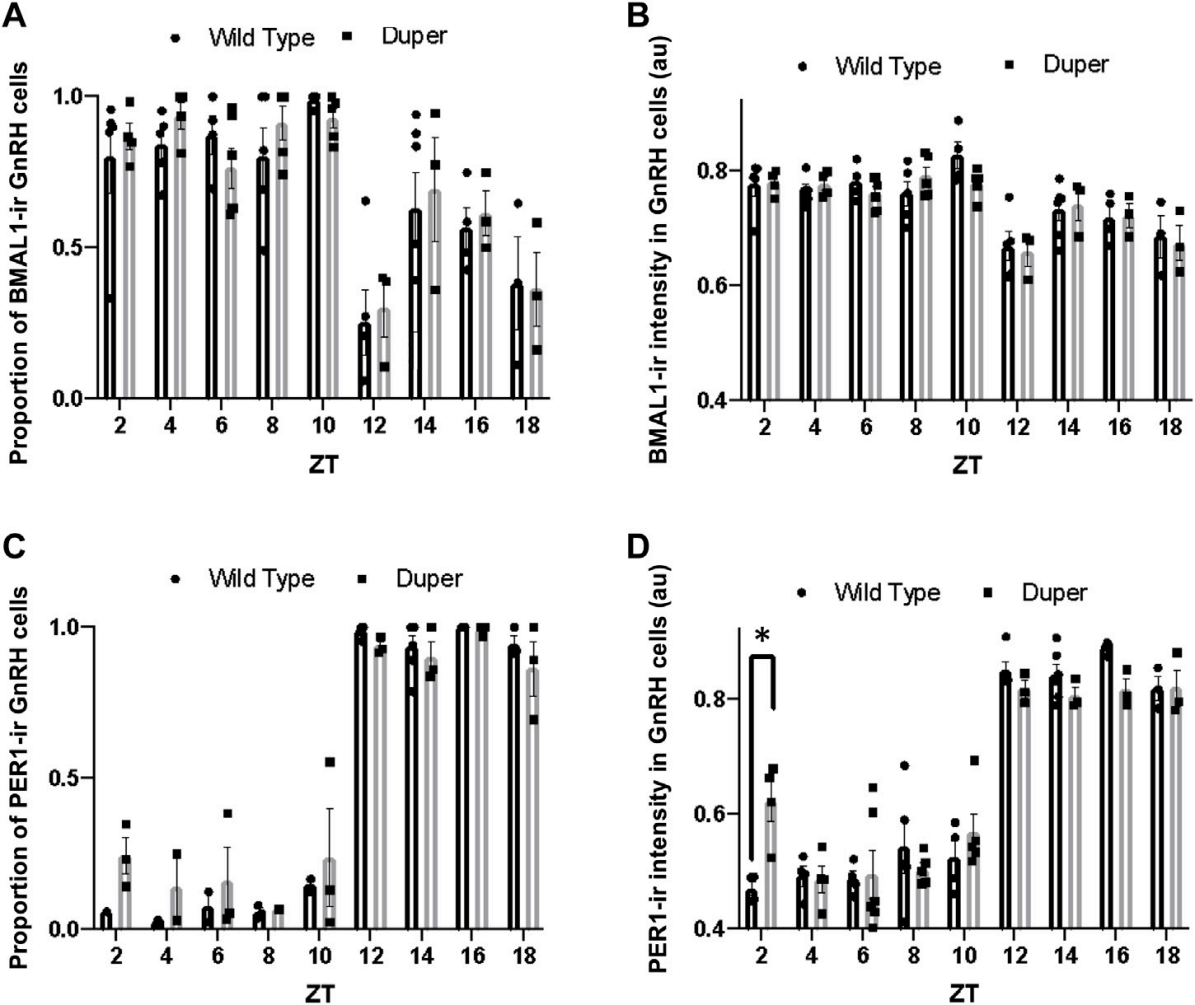
Quantification of immunofluorescence for BMAL1 and PER1 in GnRH neurons on proestrus in duper and wild-type hamsters reveals earlier onset of PERI in duper hamsters. **(A)** Proportion of BMAL1-positive GnRH cells for wild-type and duper hamsters across zeitgeber sampling times (black bars and gray bars, respectively. **(B)** Mean BMAL1 intensity in GnRH cells for wild-type and duper hamsters. **(C)** Proportion of PERI- positive GnRH cells for wild-type and duper hamsters. **(D)** Mean PER1 intensity in GnRH cells for wild-type and duper hamsters. Bar graphs represent mean ± SEM. *, *p* < 0.05.

Both the proportion of BMAL1-immunoreactive GnRH cells and the mean BMAL1-ir intensity differed with time of day (H (18) = 46.03, *p* < 0.001 and (H (18) = 43.13,*p* < 0.001; respectively; Figures 4A, B). However, Dunn’s multiple comparisons revealed no significant difference between wild type and duper hamsters in the proportion of BMAL1-immunoreactive GnRH cells or mean BMAL1 intensity in GnRH cells at any time point. Both duper and wild-type hamsters showed an approximately 1.3 fold peak-to-trough ratio of mean BMAL1 intensity measured in GnRH cells (Figure 4B).

The proportion of GnRH cells that were PER1-immunoreactive differed with time of day (H = 44.07, *p* < 0.001, Kruskal-Wallis test), but there were no significant differences between genotypes at any time point (Dunn’s multiple comparisons; Figure 4C). The mean PER1 intensity in GnRH cells also varied with time of day (H = 60.51, *p* < 0.001). Furthermore, the mean PER1 intensity levels in GnRH cells at ZT 2 was significantly higher in duper hamsters than in wild type hamsters (Z = 3.79, *p* < 0.01) (Figure 4D). The mean PER1 intensity in GnRH cells did not differ between genotypes at later times of day.

#### Descending projections of GnRH cells

**FIGURE 5:**
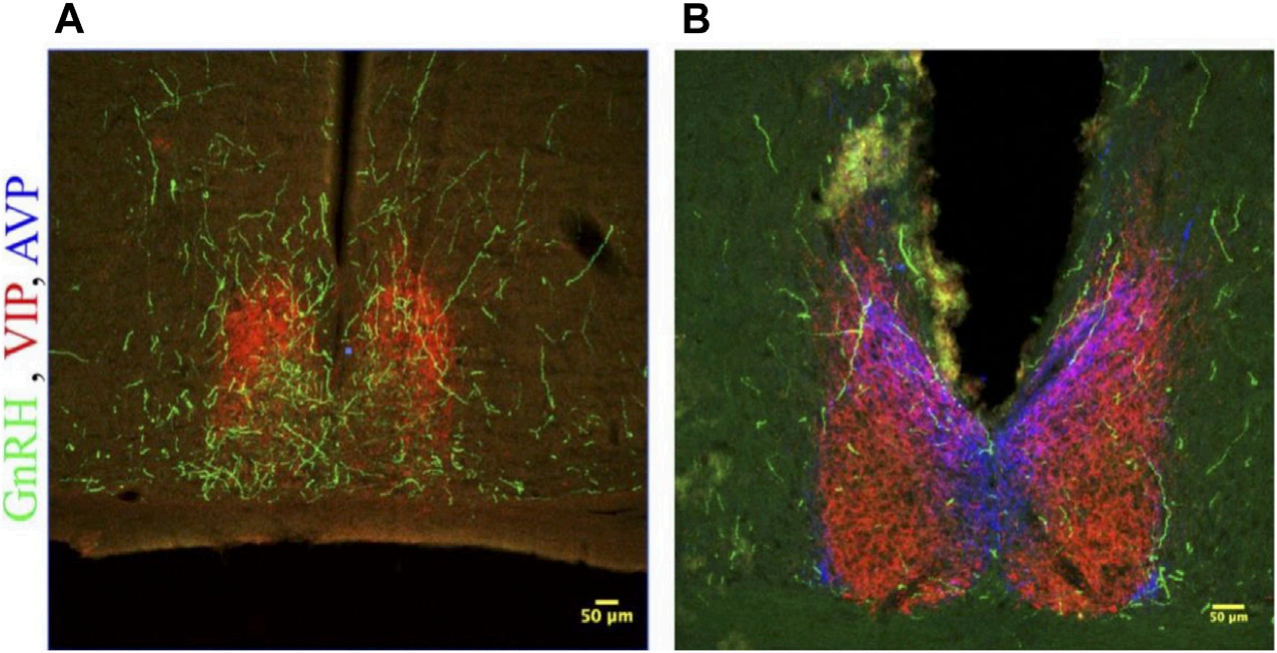
GnRH fibers traverse the SCN of both male and female hamsters. **(A)** SCN of female hamster sacrificed on proestrus at ZT8.5. **(B)** SCN of male hamster sacrificed at ZT8.5. 650x confocal z-stack images for both sexes show sub 1 micron appositions between GnRH fibers containing GnRH varicosities (Green) and SCN structures. GnRH (Green), VIP (Red), AVP (Blue) at 200x and 650x.

**FIGURE 6:**
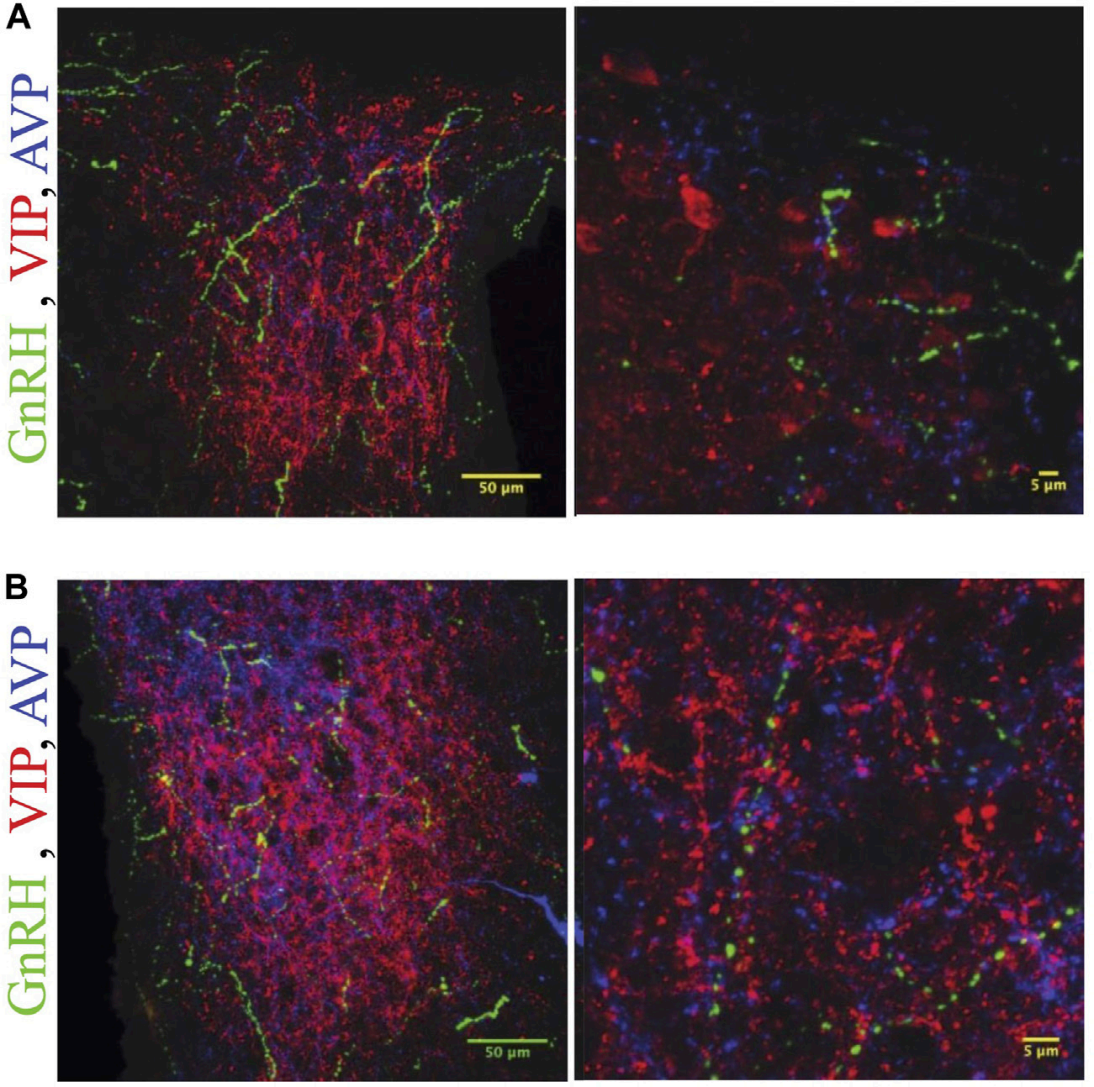
GnRH fibers are most extensive in rostral SCN of proestrous hamsters. Micrographs showing rostral **(A)** and caudal **(B)** SCN sections stained for GnRH (green), VIP (red), and AVP (blue). Higher magnification image at right illustrates appositions. GnRH fibers are more extensive in rostral than caudal SCN.

**FIGURE 7:**
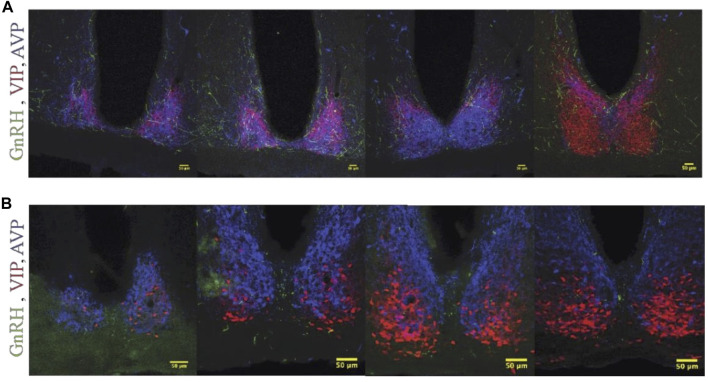
GnRH fiber projections in the SCN region are not restricted to proestrus and differ between hamsters and mice. **(A)** Female Syrian hamster sacrificed on metestrus at ZT8.5. GnRH fibers (Green), VIP (Red), AVP (Blue). GnRH fibers overlap with SCN structures. **(B)** Proestrous female C57bl/6 mouse; a few GnRH fibers are detected within the SCN, but more travel in the midline between the two SCN nuclei and appear as dots in the coronal plane. Left to right, rostral to caudal sequence.

GnRH fibers were observed coursing through the SCN of both wild type and duper hamsters. We observed appositions of GnRH fibers with both AVP and VIP-ir cells in both male and female Syrian hamsters (Figure 5). GnRH fiber density was most conspicuous in the rostral portion of the SCN (Figure 6). GnRH axons appeared as long fibers with varicosities, some on the border the SCN with a few axons traversing the entire hemisphere. GnRH fibers were also observed in the medial SCN, adjacent to the third ventricle (Figure 6). For quantitative analysis we divided the hamster SCN into dorsal and ventral regions based on AVP and VIP staining pattern and presence of cell bodies. GnRH fiber lengths measured in coronal sections of dorsal SCN (32.4 ± 2.4 μm, mean ± SEM; range 6.9 μm–87.4 μm) were not different from ventral SCN (32.2 ± 3.6 μm; 3.54 μm–138.6 μm). Consistent with findings in rats (Hoffman and Gibbs, 1982; van der Beek et al., 1997a), we observed GnRH fibers to course underneath the third ventricle and above the optic chiasm in between the two SCN hemispheres. We also found GnRH fibers in the SCN of metestrus hamsters and in males perfused at ZT8, similar to the pattern in proestrus females (Figure 6). The presence of GnRH fibers was also confirmed within the SCN of female mice (Figure 7). However, we did not find GnRH fibers in apposition with SCN cells in mice, and instead observed these fibers in the midline between the left and right SCN as a bundle in the rostral-caudal plane, orthogonal to the coronal plane of section.

### Experiment 2

#### Rate of Re-entrainment of LH surge in duper mutant and wild type hamsters

To determine whether the LH surge shifts equally rapidly in duper hamsters, we subjected female duper and wild-type hamsters to an 8-h phase advance on proestrus and characterized the LH surge 4 days later, i.e., on the next predicted day of proestrus (Figure 1A). As we found for male hamsters (Sisson et al., 2022), duper mutants re-entrained locomotor activity more quickly than did their wild type controls (Figure 1B). As was the case in stably entrained hamsters, the effects of the duper mutation on the phase of the LH surge corresponded to the behavioral changes.

**FIGURE 8:**
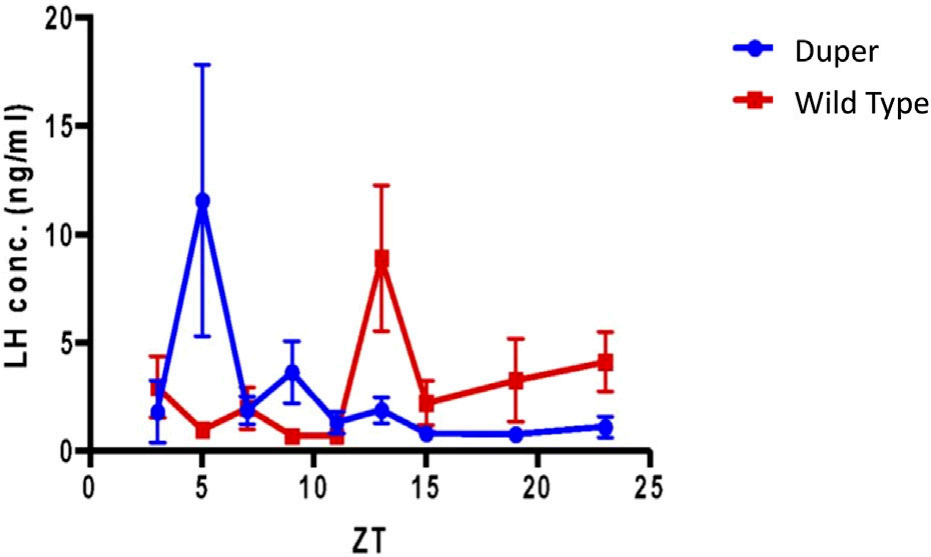
Pre-ovulatory LH surge in ovary intact duper hamsters shifts rapidly in response to an 8 hour phase advance compared to wild-types. Design of experiment 2 and actograms of representative wild type and mutant hamsters are shown in Figure 1. Mean (+/− SEM) serum LH concentrations of duper and wt hamsters are shown in blue and red, respectively. Values differ at Zeitgeber Time (ZT) 5 (*p* < 0.05) and ZT 13 (*p* < 0.01).

Over the 4 days after the phase advance of the LD cycle, the mean onset of locomotor activity of wt hamsters shifted by 3.5 ± 0.7 h. The mean time of the last locomotor onset was 3.9 ± 0.5 h after the time of lights out (i.e., ZT 15.9). The timing of the LH surge was variable among wild type hamsters, and none of these animals displayed a surge (defined as LH concentration > 6 ng/mL) at the expected time for the new LD cycle (between ZT 7 and ZT 9; Figure 8). Three of 7 wild type hamsters tested at ZT 13 displayed an LH surge. Baseline levels of LH were elevated at ZT 3, 7, 15, 19, and 23.

In contrast, the phase of activity onset shifted by 6.9 + 0.9 h within 4 days of the 8 h advance of the LD cycle in duper hamsters (*p* < 0.005 vs. wild types). Locomotor activity began 1.5 + 0.2 h before lights off (i.e., ZT10.5; *p* < 0.001 vs. wild types). The LH surge occurred at ZT5, similar to that of unshifted duper hamsters in experiment 1. Thisindicates that the phase advance of both locomotor activity and the LH surge had largely been completed within one estrous cycle in the mutants. Three of 7 duper hamsters demonstrated a LH surge at ZT 5 (Figure 8). With the exception of ZT 9, at which 2 of 6 duper hamsters demonstrated elevated LH levels (>6 ng/mL), LH levels returned to baseline at the other time points tested. Kruskal-Wallis test revealed a significant difference in LH levels between genotypes [H (18) = 37.57, *p* < 0.01]. Dunn’s multiple comparisons showed that LH levels at ZT 5 of the new LD cycle was significantly higher in duper hamsters (7.07 ± 2.96 ng/mL, *n* = 9) than in wild type hamsters (0.94 ± 0.30 ng/mL, *n* = 7; Z = 2.79, *p* = 0.05). The difference between control and mutant hamsters was reversed at ZT 13, when wild type hamsters (7.27 ± 2.24 ng/mL, *n* = 7) had higher LH levels than in duper hamsters (0.99 ± 0.42 ng/mL, *n* = 5; Z = 2.77, *p* = 0.05). Thus the LH surge shifts more rapidly in duper hamsters than in wild-type hamsters.

### Experiment 3

#### Photoperiodic responses in duper and wild type hamsters

**FIGURE 9:**
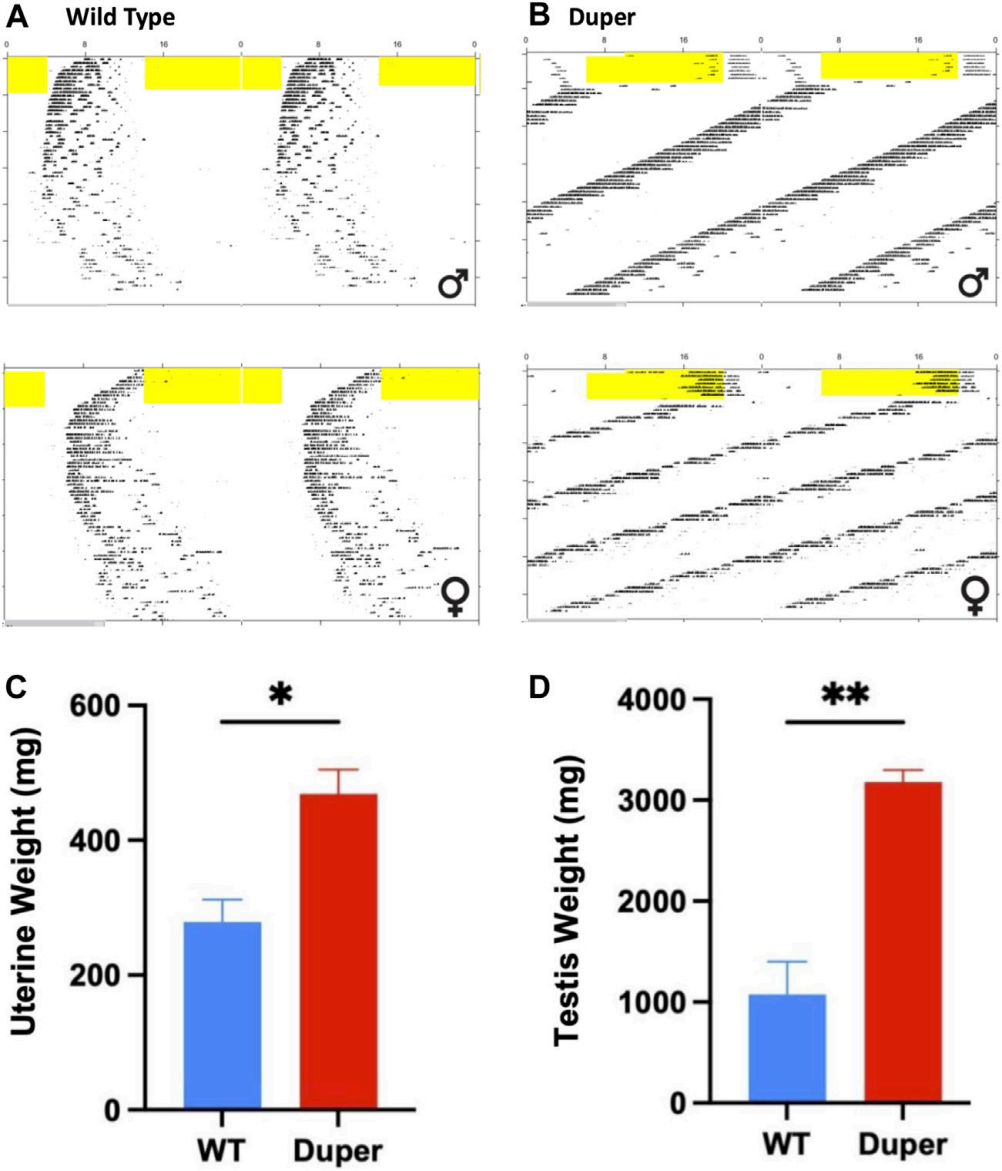
Maintenance in 8 weeks of constant darkness in Experiment 3 arrested reproducitve function in wild type, but not duper mutant hamsters. Top: Double plotted actograms of wild type **(A)** and duper mutant **(B)** hamsters subjected to an 8 h phase advance and released into DD 10 days later. Yellow shading indicates light phase. Note that wild type hamsters are slow to re-entrain after the shift. In DD, duper female hamster shows scalloping, indicating persistence of the estrous cycle. Wild type hamsters exhibit progressive disorganization of the free running behavioral rhythm, but dupers do not. Bottom: Mean (+SEM) uterine **(C)** and paired testis **(D)** weights at time of sacrifice after 8 weeks of DD.

The duper mutation prevented both the disruption of locomotor activity rhythms and gonadal regression observed in wt hamsters maintained for 8 weeks in DD. As expected, the free running period of duper hamsters was significantly shorter than that of wild types (23.10 ± 0.09 h vs. 24.07 ± 0.07 h, mean ± SEM; *p* < 0.001; Figures 9A, B). As previously described (Ellis and Turek, 1983), free running behavioral rhythms of wt hamsters became less robust with protracted exposure to DD. This effect was not evident in duper mutants. The precision of free running rhythms of wild types during the last 2 weeks of DD, assessed as error of the linear regression fit of onsets, was significantly lower in wild types than dupers (1.19 ± 0.43 vs. 0.29 ± 0.04 h; *p* < 0.05).The total activity (number of wheel revolutions) over the last 2 weeks of DD was greater in duper mutants (3,793 ± 390 revolutions) than in wild types (1,596 ± 550; *p* < 0.01).

At the time of collection, paired testis weights of duper hamsters exceeded those of wild types (3.18 ± 0.13 vs. 0.47 ± 0.04 g, respectively, *p* < 0.001; Figure 9C). Among females, uterine weights of dupers were more than 3-fold greater than those of wt (1.07 ± 0.36 vs. 0.28 ± .04 g, respectively; Figure 9D).

Serum melatonin concentrations were similar in late subjective night among wt and duper hamsters housed for 8 weeks in DD (9.5 ± 4.3 pg/mL in duper vs. 11.6 ± 4.8 pg/mL in wt hamsters; *n* = 14 and *n* = 10, respectively). For comparison, wt and mutant hamsters were sacrificed in 14L:10D at approximately ZT19. Serum levels of melatonin tended to be lower in dupers than in wt (18.3 ± 4.0 vs. 31.3 ± 10.3 ng/mL, respectively; n = 5 hamsters/group). Daytime melatonin concentrations were uniformly below 1.5 ng/mL.

## Discussion

Successful reproduction requires temporal coordination of events at the brain, pituitary, and gonadal levels. It has long been apparent that a circadian clock underlies both the timing of the LH surge (Everett and Sawyer, 1950; Legan and Karsch, 1975; Swann and Turek, 1985; de la Iglesia et al., 2003) and photoperiodic time measurement (Elliott et al., 1972) in muroid rodents. Our findings on the duper mutant hamster implicate *Cry1*, a core component of the cell autonomous circadian clock, in timing of the LH surge and photoperiodic responses. For both estrous cycles and photoperiodism, the loci at which *Cry1* expression exerts its influence remain to be determined.

Little is known about the mechanisms that ensure stability of the phase of the LH surge or persistence of estrous or menstrual cycles when schedules change. Jet lag and shift work impact the lives of millions of people, and women experience irregular menstrual cycles under these circumstances (Mahoney, 2010; Komada et al., 2018; Yaw et al., 2020). In mice, the timing of the LH surge changes only gradually after the LD cycle is shifted (Bahougne et al., 2020). Presumably, circadian clocks at multiple levels, including the brain’s surge circuit, the pituitary, and the ovaries must all shift to ensure phase relationships required for coordinated function. It is remarkable that the phase of behavioral rhythms and that of the LH surge appear to shift on a similar time scale in dupers (Figure 8), as the former is controlled by humoral outputs and the latter by axonal projections of the pacemaker (Silver et al., 1982; de la Iglesia et al., 2003). The slow rate of re-entrainment in wild types may result at least in part from delays in re-alignment of circadian clocks at different brain loci, hypophyseal, and ovarian levels. The acceleration of re-entrainment of the LH surge in duper hamsters may reflect a role of *Cry1* as a rate-limiting step in the re-establishment of phase coherence among multiple oscillators. Nevertheless, careful examination of numbers of ova shed and fecundity may reveal deficits in reproduction upon phase shifts even in duper hamsters.

Estrous cycles are irregular in both *Clock* mutant and *Bmal1*-deficient mice (Miller et al., 2006; Chu et al., 2013). Nevertheless, these animals can reproduce even when the LH surge is of reduced amplitude, mis-timed, or even absent. Deletion of *Cry1* and *Cry2*, alone or in combination, does not block female reproductive function in mice. We find that estrous cycles are quite regular in the *Cry1*-deficient duper mutants (present study; Bahiru and Bittman, 2023). This is consistent with observations of Takasu et al. (2015) on Cry1-deficient mice, although the onset of irregular estrous cycles may occur at an earlier age in such animals and entrainment to LD cycles whose period (T) approximates τ_DD_ can ameliorate female reproductive function. Examination of the precise *timing* of the LH surge provides further evidence of circadian control of ovulation. In ovary-intact, wild type hamsters, the LH surge occurs 2-3 h before the onset of activity (Stetson and Gibson, 1977). When ovariectomized hamsters are treated with estrogen, daily surges occur at circadian intervals (Norman and Spies, 1974; Swann and Turek, 1985; de la Iglesia et al., 2003). Evidence that the free running period of these daily LH surges is reduced in *tau* mutant hamsters (Lucas et al., 1999) indicates the role of the TTFL in controlling GnRH discharge, but the phase of the surge (approximately circadian time 7) appears to be similar in wt and *tau* mutant hamsters. In contrast, we observed that the LH surge of stably entrained ovary-intact duper hamsters occurs earlier in the day than it does in wt. While this is consistent with the effect of mutations that shorten circadian period to advance the phase angle of entrainment of rhythms of locomotor activity, it suggests that mutations which impact different components of the negative limb of the TTFL vary in their impact on timing of the preovulatory LH surge.

It is possible that circadian regulation of reproduction can be accounted for solely at the level of the SCN (Rusak and Morin, 1976; Brown-Grant and Raisman, 1977; Wiegand and Terasawa, 1982). It seems more likely, however, that circadian clocks may regulate the LH surge in multiple cell types and at several loci (Palm et al., 2001; Bittman, 2019). Vasopressinergic cells in the dorsomedial “shell” of the SCN likely regulate both the LH surge and peripheral clocks (Palm et al., 2001; Miller et al., 2004; Miller et al., 2006; Jamieson et al., 2021). Such efferents regulate kisspeptinergic neurons of the AvPV, which in turn provide critical input to GnRH cells in order to trigger the LH surge (van der Beek et al., 1997a; Vida et al., 2010). VIPergic projections from the SCN to the GnRH cells, or to RFRP-3 cells of the dorsomedial hypothalamus (Williams et al., 2011; Russo et al., 2015), may also contribute to clock control of GnRH cells. Recently, prokineticin 2 has been implicated as an important component of SCN timekeeping and a potential regulator of GnRH discharge (Xiao et al., 2014; Morris et al., 2021). Experimental strategies to examine effects of selective deletion of *Cry1* in either the AVP, VIP and/or PK2 cells of the SCN may help to explain effects of the duper mutation on either the timing of the surge in stably entrained animals or the latency with which the phase of LH secretion shifts when the LD cycle is advanced. *Cry1* expression may also be critical in cell types within the LH surge circuit that lie outside the SCN. Conditional knockout of *Cry1* in kisspeptin cells of the AvPV and/or arcuate nucleus, in RFRP-3 neurons of the DMH, and/or in the GnRH cells themselves may also help to determine sites at which this core clock gene influences phase of the LH surge and/or the rate at which its timing can shift (Hickok and Tischkau, 2010; Xu et al., 2011; Smarr et al., 2013; Bittman, 2019).

Operation of the TTFL within GnRH cells may also contribute to the timing and amplitude of their activation on the afternoon of proestrus (Williams et al., 2011). We observed antiphase expression of PER1 and BMAL1 in GnRH cells of both wild type and mutant female hamsters. Although this result is similar to findings of Hickok and Tischkau (2010) on antiphase expression of PER2-ir and BMAL1-ir in GnRH-eGFP neurons in female mice, we found relatively little change in BMAL1-ir intensity across sampling times. This result is consistent with findings in cell lines that nuclear concentrations and diffusion rates of BMAL1 remain relatively consistent through day and night, despite rhythmicity of interactions with other core clock proteins (Koch et al., 2022). Although patterns of PER1-ir and BMAL1-ir were generally similar between duper and wt, PER1-ir intensity was significantly higher in mutants at ZT2. This may be relevant to the advanced phase of the LH surge in duper mutants (Figure 2). Sampling at 2 h intervals, while intensive, may be insufficient to characterize fully the pattern of clock gene expression in GnRH cells. Furthermore, clock proteins other than PER1 may be more strongly impacted by the duper mutation.

Effects of the duper mutation on hypophyseal and ovarian function may also contribute to changes in the phase of the LH surge and/or the rate of its re-entrainment. Conditional deletion of *Bmal1* in gonadotropes reduces the regularity of mouse estrous cycles (Chu et al., 2013). Deficiency of *Bmal1* in ovarian theca cells impairs fertility and alters expression of LH receptor and thus gonadal steroidogenesis and gametogenesis (Liu et al., 2014; Mereness et al., 2016; Jiang et al., 2022). Thus clock function in the ovary may contribute indirectly to determination of the timing of ovulation, as estrogen and progesterone regulate the phase and amplitude of the LH surge (DePaolo and Barraclough, 1979; Stephens et al., 2015; Leite et al., 2016).

Models of circadian control of the LH surge commonly place the circadian pacemaker upstream of the GnRH effector cells. While it is likely that SCN efferents ensure phase coherence of subordinate circadian oscillators in GnRH cells, our finding that GnRH fibers course through the hamster SCN suggests descending modulation of the surge by the SCN. Our observation is consistent with earlier reports in rats (Hoffman and Gibbs, 1982; van der Beek et al., 1997b) and hamsters (Morin, 2013), and adds the finding that GnRH fibers make appositions with both VIP and AVP-ir neurons in the SCN. The idea that GnRH “dendrons” allow input to descending GnRH fibers approaching the median eminence opens the possibility that similar contacts in the SCN might gate conduction of action potentials (Herbison, 2021). Alternatively, such appositions may inform the SCN of the progress of the surge or the general status of GnRH discharge. Further insight into what information (if any) might be exchanged will require ultrastructural studies. We find that such appositions are also detected on metestrus and in male hamsters. This discourages speculation that such descending contacts have a specific role in coordinating the LH surge. It remains possible that communication between GnRH fibers and SCN elements plays a more general role in circadian coordination of gonadotropin release, i.e., one that is not specific to the sexually differentiated LH surge. We did not examine clock protein staining in GnRH cells on other days of the hamster estrous cycle, but the pattern of GnRH fiber staining in the SCN was similar on metestrus and proestrus.

Our finding that both male and female duper hamsters maintain reproductive capacity after 8 weeks of DD implicates *Cry1* in regulation of seasonal reproductive rhythms. Given the circadian basis of photoperiodic time measurement, it is expected that seasonal responses will be altered in mutants that lack TTFL components. Nevertheless, the role of different core clock genes may differ. The *tau* mutation does not eliminate the hamster photoperiodic response; unlike dupers, these period mutants regress their gonads in DD (Loudon et al., 1998). Nevertheless, the critical night length for testicular regression and suppression of prolactin secretion is shortened in tau mutants, while the entrained phase of onset of melatonin secretion is advanced and its duration is extended (Stirland et al., 1995; Stirland et al., 1996a; Stirland et al., 1996b; Lucas et al., 1999). Assessment of the photoperiodic response in period mutants is complicated by the fact that the phase angle of entrainment is altered, so that animals competent to respond may nevertheless photostimulate in a T24 cycle (Shimomura et al., 1997). Similarly, arrest of estrous cycles in Cry1-deficient mice held on T24 are eliminated when they are maintained in an T cycle whose period matches that of their endogenous clock (Takasu et al., 2015). In the present experiments, we maintained duper hamsters in DD rather than short photoperiods in order to avoid any such effects. Thus the failure of the reproductive system of duper hamsters to regress cannot be attributed to changes in the entrained phase angle but rather indicate a role of CRY1 in the response to light deprivation. It will be necessary to examine the response of duper hamsters to short photoperiods in different T cycles in order to rule out the possibility that the gonads can regress when the phase angle of entrainment is normalized, but our finding that they do not respond to DD make this unlikely.

As for the LH surge system, the loci at which the duper mutation affects photoperiodic responses remain to be determined. *Cry1* may be necessary for responses to daylength at the level of the SCN, in which multiple oscillators vary their phase relationship as the interval between dawn and dusk changes (Schwartz et al., 2001; Inagaki et al., 2007; Lucassen et al., 2012; Tackenberg and McMahon, 2018; Gupta et al., 2013). Wild type hamsters decreased activity levels over the course of maintenance in DD, and running onsets became irregular as the gonads regressed. These changes were absent in duper mutants, suggesting a role of *Cry1* in photoperiodic responses at the level of the SCN. Exercise retards or reverses reproductive arrest and metabolic changes in hamsters exposed to short days (Borer et al., 1983; Scherbarth et al., 2008). Thus the effect of duper to maintain robust locomotor activity may contribute to persistence of reproductive function in DD. While some changes in circadian function may be secondary to withdrawal of sex steroid secretion upon gonadal collapse, short days reduce the effect of testosterone to restore wheel running in castrated hamsters (Ellis and Turek, 1983). However, photoperiod can alter SCN organization and function in melatonin-deficient mouse strains and are thus likely to be pineal independent (Evans, et al., 2013; Mendoza-Viveroset al., 2017; Tackenberg and McMahon, 2018).

Clock gene expression in the pineal gland may also regulate melatonin production (Mays et al., 2011). Melatonin synthesis in C3H mice is increased in Per1-deficient but severely reduced in Cry1/Cry2 double knockout strains (Christ et al., 2010; Yamanaka et al., 2018). These effects may reflect clock gene control of AANAT transcription not only by control of rhythms of sympathetic input, but also via regulation of E-box elements and the response to β-adrenergic activation within the pineal gland, respectively. In tau mutant Syrian hamsters, the critical duration of melatonin signals and the frequency with which they must be presented in order to induce reproductive arrest are altered (Stirland et al., 1996b). We measured serum melatonin concentrations in wt and duper hamsters to begin to address the question of whether the mutation affects pineal output. Our results demonstrate the competence of the duper hamster to secrete melatonin in DD, although the timing and duration of melatonin production in Cry1-deficient hamsters under free running or entrained conditions remain to be examined. The duration and amplitude of rhythms of melatonin secretion change with protracted exposure to DD (Elliott and Tamarkin, 1994). This could contribute to the lower levels of melatonin we detected DD hamsters relative to those entrained to 14L:10D. Further experiments will be needed to characterize melatonin secretion across a range of ZTs and photoperiods, and to determine whether the response of duper hamsters to photoperiod differs from the wild type.

In addition to the role of clock function in the adenohypophysis to regulate gonadotropin and prolactin secretion, circadian oscillators in the pituitary complex are critical to seasonal changes in reproduction. The phase relationship between clock proteins in the *pars tuberalis* may lie at the heart of an internal coincidence system that determines responses to melatonin duration and thus to photoperiod (Lincoln et al., 2002; Johnston et al., 2006; Dupré et al., 2008; Wood et al., 2020). Melatonin regulates expression of *Cry1* and associated genes in ovine *pars tuberalis* (Dupré et al., 2008). Although such a system is little explored in hamsters, *Cry1* function in the *pars tuberalis* may be critical in this long day breeder as well. Future experimental strategies to examine effects of the duper mutation on expression of melatonin-regulated gene expression, including *Eya3* and *TSH*, will help to clarify the role *of Cry1* in seasonal reproduction.

Our findings advance our understanding of environmental control of neuroendocrine function. Further work in Syrian hamsters can capitalize on the robust estrous cycle, highly regular circadian system, and strong photoperiodic response in order to clarify both the molecular mechanisms and anatomical loci through which circadian oscillators regulate reproduction.

## Data Availability

The raw data supporting the conclusion of this article will be made available by the authors, without undue reservation.
